# Plaque Size Is Decreased but M1 Macrophage Polarization and Rupture Related Metalloproteinase Expression Are Maintained after Deleting T-Bet in ApoE Null Mice

**DOI:** 10.1371/journal.pone.0148873

**Published:** 2016-02-17

**Authors:** Aikaterini Tsaousi, Elaine M. Hayes, Karina Di Gregoli, Andrew R. Bond, Laura Bevan, Anita C. Thomas, Andrew C. Newby

**Affiliations:** School of Clinical Sciences, University of Bristol, Bristol, United Kingdom; Brigham and Women's Hospital, Harvard Medical School, UNITED STATES

## Abstract

**Background:**

Thelper1 (Th1) lymphocytes have been previously implicated in atherosclerotic plaque growth but their role in plaque vulnerability to rupture is less clear. We investigated whether T-bet knockout that prevents Th1 lymphocyte differentiation modulates classical (M1) macrophage activation or production of matrix degrading metalloproteinases (MMPs) and their tissue inhibitors, TIMPs.

**Methods & Results:**

We studied the effect of T-bet deletion in apolipoproteinE (ApoE) knockout mice fed a high fat diet (HFD) or normal chow diet (ND). Transcript levels of M1/M2 macrophage polarization markers, selected MMPs and TIMPs were measured by RT-qPCR in macrophages isolated from subcutaneous granulomas or in whole aortae. Immunohistochemistry of aortic sinus (AS) and brachiocephalic artery (BCA) plaques was conducted to quantify protein expression of the same factors.

Deletion of T-bet decreased mRNA for the M1 marker NOS-2 in granuloma macrophages but levels of M2 markers (CD206, arginase-1 and Ym-1), MMPs-2, -9, -12, -13, -14 and -19 or TIMPs-1 to -3 were unchanged. No mRNA differences were observed in aortic extracts from mice fed a HFD for 12 weeks. Moreover, AS and BCA plaques were similarly sized between genotypes, and had similar areas stained for NOS-2, COX-2, MMP-12 and MMP-14 proteins. T-bet deletion increased MMP-13, MMP-14 and arginase-1 in AS plaques. After 35 weeks of ND, T-bet deletion reduced the size of AS and BCA plaques but there were no differences in the percentage areas stained for M1 or M2 markers, MMPs-12, -13, -14, or TIMP-3.

**Conclusions:**

Absence of Th1 lymphocytes is associated with reduced plaque size in ApoE knockout mice fed a normal but not high fat diet. In either case, M1 macrophage polarization and expression of several MMPs related to plaque instability are either maintained or increased.

## Introduction

Atherosclerosis and in particular plaque rupture underlie myocardial infarctions and strokes, which remain the greatest cause of mortality in developed countries, and are increasing in prevalence worldwide (https://apps.who.int/infobase/mortality.aspx). Atherosclerosis is a slowly-progressing, chronic inflammatory disease that is initiated by deposition of cholesterol-rich lipoproteins in the artery wall. Modification of these lipids then leads to production of cytokines and chemokines that recruit monocytes, which differentiate into macrophages under the influence of colony stimulating factors (CSFs) [1]. Mature macrophages express a variety of scavenger receptors that promote lipid uptake leading to the formation of foam cell macrophages (FCMs). The key role of macrophages in atherosclerosis is demonstrated by their abundance in human plaques (mostly as FCMs) and by the dramatic reduction in atherosclerosis after genetic [1] or pharmacological [2] deletion of macrophages in mice. Modified lipids can stimulate the innate immune system into producing cytokines, pathogen associated molecular patterns and other alarmins that together promote classical macrophage activation [3]. Classically activated (otherwise known as M1) macrophages produce high levels of toxic reactive oxygen intermediates, including nitric oxide (NO), by upregulating the inducible nitric oxide synthase, NOS-2. They also generate pro-inflammatory prostaglandins through cyclooxygenase-2 (COX-2). Moreover, M1 macrophages overexpress several matrix metalloproteinases (MMPs) that are directly implicated in plaque rupture [4]. Amongst these, collagenases may be particularly important because they degrade the strength-giving component of fibrous plaque caps [5]. Classically activated macrophages over-expressing the M1-related MMPs are abundant in the vulnerable shoulder regions of human atherosclerotic plaques [4]. Finally, several factors that reduce morphological surrogates of plaque instability (notably knockout of CD40 ligand [6]) also decrease markers of classical activation in mouse plaques. Strategies that would reduce or reverse M1 polarization might therefore prevent plaque ruptures and reduce the incidence of myocardial infarctions.

New epitopes generated by lipid modification can also trigger the adaptive immune system; there is an impressive volume of experimental evidence that innate and adaptive immune systems cooperate in perpetuating plaque progression [7]. For example, T-lymphocytes are present in atherosclerotic plaques at all stages in humans [8, 9] and rodents [10, 11], with CD4+ Thelper (Th)-lymphocytes more abundant than CD8+ cytotoxic T-cells [12]. Furthermore, T-cell clones reactive against relevant antigens, including native and modified lipoproteins [13] and heat shock protein 60 [14], have been isolated from human plaques. In consequence, immunomodulation has been the subject of preclinical studies and clinical trials as a way to reduce atherosclerosis [15]. On the other hand, Rag-1 or Rag-2 gene deletion, which depletes all T- and B-lymphocytes has little influence on plaque progression in susceptible mice strains, especially in the presence of a high fat diet (HFD) [16]. Furthermore, the percentage of FCMs expressing M1 marker genes, MMPs and TIMPs is also unaffected by Rag-1 knockout [16]. However, these results might arise because opposing stimulatory and inhibitory actions of different T- and B-cell subsets lead to a neutral response overall.

CD4^+^ T-lymphocytes are categorized into subsets according to their functions and the spectrum of cytokines they produce [17]. For example, Th1 cells can promote classical macrophage activation partly by producing interferon-γ (IFNγ), whereas Th2 cells down regulate Th1-mediated responses and provoke allergy, in part through secreting interleukin (IL)-4, -5, -10 and -13 [18]. T-cell mediated immune responses in atherosclerosis are believed to be Th1 dominated, based on evidence that IFNγ but not IL-4 is abundant in human plaques [19]. Severe hypercholesterolemia is also associated with a Th1/Th2 switch in T-lymphocytes isolated from atherosclerosis-susceptible ApoE knockout (EKO) mice [20]. A role for Th1 cells in promoting atherosclerosis has been inferred because mice that are deficient in either IFNγ receptors [21] or IFNγ itself [22, 23] have smaller lesions, whereas administration of exogenous IFNγ to EKO mice augments atherosclerosis [24]. Differentiation of each of the Th-lymphocyte subsets is orchestrated by a distinct master regulator transcription factor [25]. Specifically, T-bet mediates IFNγ production from CD4^+^ T-cells [26, 27] and dendritic cells [28], which promotes Th1 and suppresses Th2 differentiation. T-bet deficiency decreases the size of diet-induced atherosclerotic plaques in male, low density lipoprotein receptor (LDLR) deficient mice [29], which further supports the role of Th1 lymphocytes in lesion progression. However, its role in plaque destabilization and rupture is less clear. T-bet deficiency does not change the proportion of macrophages in plaques from LDLR deficient mice; and although it decreases the proportion of smooth muscle cells, this may simply reflect the less advanced state of plaque development [29].

To come to a definite conclusion whether Th1 lymphocytes contribute to M1 macrophage activation and plaque destabilization *in vivo* [30], we investigated the impact of T-bet deletion on M1 and M2 polarization markers and MMP/TIMP production in FCMs from EKO mice. We studied steady-state mRNA levels in FCMs isolated from subcutaneous granulomas and in the whole aortae of mice fed a HFD. We also examined advanced plaques in the aortic sinus (AS) and brachiocephalic artery (BCA) using histological morphometry in mice fed a HFD or normal chow diet (ND).

## Materials and Methods

### Breeding of ApoE/T-bet double knockout mice and harvesting of tissues

ApoE knockout mice (B6.129P2-*Apoe*^*tm1Unc*^/J) and T-bet knockout mice (B6.129S6-Tbx21^*tm1Glm*^/J) on a C57Bl6 background were purchased from The Jackson Laboratory and mated together. The doubly heterozygous progeny were inter-crossed to generate double knockout T-bet^-/-^ApoE^-/-^ (DKO) mice and T-bet^+/+^ApoE^-/-^ (EKO) controls. Genotypes were confirmed via PCR: tail or ear tissue from each mouse was digested overnight in Direct PCR-tail solution (Bioquote, UK) and proteinase K (Sigma, UK). PCR was performed on the digested tissue using Crimson Taq Pol (New England Biolabs, UK) with dNTPs from Bioline (UK) and primers designed by The Jackson Laboratory (Table 1).

**Table 1:** Genotyping primers.

Primer Name	Primer Sequence
ApoE Common	GCCTAGCCGAGGGAGAGCCG
ApoE WT	TGTGACTTGGGAGCTCTGCAGC
ApoE KO	GCCGCCCCGACTGCATCT
T-bet Common	GCGCGAAGGGGCCACCAAAGAACGGAG
T-bet WT	GACTGAAGCCCCGACCCCCACTCCTAAG
T-bet KO	TGGGCATACAGGAGGCAGCAACAAATA

Mice were either fed a normal chow diet (ND: 3.5% fat, LabDiet UK) until 35 weeks old or were given a high-fat diet (HFD: 21–23% fat, Special Diet Services, UK) for 12 weeks starting at 5 weeks of age. The mice were given an overdose of halothane (to prevent monocyte/macrophage mobilisation) and their tissues were harvested either immediately or after perfusion with phosphate buffered saline (PBS) followed by 10% formalin/PBS (v/v) at physiological pressure. Tissues harvested included tail (for genotyping), blood (cardiac puncture), spleen, thymus, liver, heart (for AS), BCA, the entire aorta and peritoneal macrophages (PBS lavage). A further set of mice were fed the HFD from 6 to 12 weeks of age and used to generate sponge FCM in vivo (see section: Generation and isolation of FCMs from subcutaneous sponges).

The housing and care of all animals and procedures used in these studies was in accordance with and under license of the Animals (Scientific Procedures) Act 1986 (London, United Kingdom), and conform to the Guide for the Care and Use of Laboratory Animals published by the U.S. National Institutes of Health (Publication No. 85–23, revised 1996). The study received local institutional review board (University of Bristol, Bristol, United Kingdom) approval. The mice were bred and maintained by the University of Bristol Animal Services Unit, and kept in isolators or scantainers and given sterile food and water *ad libitum*.

### Peripheral blood mononuclear cell (PBMC) isolation and differentiation

Pooled peripheral blood was collected by cardiac puncture from C57Bl6 mice with heparin as anti-coagulant and diluted with PBS without calcium and magnesium (Lonza, UK) (ratio 1:1). The diluted samples were subjected to density gradient separation on Ficoll Paque Plus (GE Healthcare Life Sciences, UK) and centrifuged. After centrifugation the PBMC layer was collected and washed in Hank's Balanced Salt Sodium (HBSS) with phenol red without calcium and magnesium (Lonza). Non-adhered mouse monocytes were positively selected from PBMC preparations using CD11b conjugated magnetic beads (Miltenyi Biotec, UK) according to the manufacturer’s instructions. The CD11b selected monocyte suspension was spun and the pellet re-suspended in fresh media and counted. To differentiate blood-derived monocytes into macrophages, PBMCs were re-suspended in RPMI 1640 media (Life Technologies, UK) supplemented with antibiotics, glutamine and 10% foetal calf serum (FCS, Invitrogen, UK) and placed in 6-well culture plates (at 5x10^5^ cells/well) and 12-well culture plates (2.5x10^5^ cells/well). After 2 h the plates were washed in RPMI/FCS to remove non-adherent cells. The remaining adherent monocytes were cultured for 6–7 days with RPMI/FCS in the presence of 40 ng/ml recombinant mouse M-CSF (Miltenyi Biotec), allowing the cells to differentiate into macrophages. At this time the cells were exposed to selected growth factors for 18 h (in FCS free media), as specified in the text. These included mouse IFNγ at 20 ng/ml (Miltenyi Biotec) and human tissue necrosis factor-α (TNFα) at 10 ng/ml (R&D Systems, USA). Human cytokines were used only when their efficacy on mouse cells had been previously documented by the suppliers.

### Assays on blood samples

Blood was taken via cardiac puncture and heparinized plasma was subsequently analysed for total, HDL and LDL/VLDL cholesterol (Cholesterol/Cholesterol Ester Quantification Kit, Abcam, UK), based on the manufacturer’s instructions. The levels of selected M1 and M2 cytokines were assessed in additional samples of mouse plasma using a Bio-Plex Pro Mouse Cytokine Th1/Th2 Panel 8-plex assay (Bio-Rad, USA), according to manufacturer’s instructions.

### Generation and isolation of FCMs from subcutaneous sponges

Mice were given the HFD from 6 weeks of age. Two weeks later and under isoflurane anaesthesia 0.5 cm^3^ sterile polyurethane sponges (Merck, UK) containing ~50 μL Matrigel (VWR, UK) were placed under the dorsal skin to generate FCMs, as described previously [31, 32]. Buprenorphine analgesic was given, and the mice were fed the HFD for a further 4 weeks before they were sacrificed using an anaesthetic overdose. Freshly recovered sponges were treated with 0.75 mL undiluted dispase (BD Biosciences, USA) and then squeezed to obtain a cellular exudate. FCMs were purified using density centrifugation on a metrizamide gradient (1.3507 refractive index, Sigma, UK) and differential adherence, as previously described [33]. Sponge FCMs were lysed immediately for RNA or protein extraction or left to adhere onto glass coverslips where their proliferative capacity was assessed via BrdU incorporation.

### BrdU immunocytochemistry (purified and cultured FCMs)

Isolated FCMs from sponges were left to adhere to coverslips overnight at 37°C. BrdU (Sigma) was added to the cultures for 8 h. The cells were then fixed with 4% paraformaldehyde in PBS (10 min at room temperature) and permeabilised with 2M HCL (30 min at 37°C) before they were incubated with anti-BrdU antibody (Sigma) at 4°C overnight. Following a PBS wash, cells were incubated with biotinylated antibody (DAKO, UK) followed by extravidin-HRP (Sigma) (30 min each at room temperature). Finally, signal was developed with 3,3′-diaminobenzidine (DAB) (Sigma).

### RNA extraction, reverse transcription and quantitative PCR

Purified FCMs were lysed in RLT solution (Qiagen Ltd, UK) with β-mercaptoethanol and total RNA was extracted using the Qiagen RNeasy kit (Qiagen Ltd), according to the manufacturer’s instructions. The quantity and quality of resulting RNA was assessed using a NanoDrop ND-1000 spectrophotometer (LabTech International, UK). A total of 100–200 ng of RNA per sample was next reverse transcribed to cDNA using QuantiTect Reverse Transcription Kit (Qiagen Ltd), according to the manufacturer’s instructions. cDNA was amplified via real time quantitative PCR performed on Light Cycler 1.5 (Roche, UK), using the QuantiTect® SYBR® Green PCR Kit (Qiagen Ltd). Most primers used were designed in-house (Table 2) and synthesized by Sigma. Copy numbers of gene transcripts per total ng RNA input were calculated using standard curves constructed from purified amplicons, with the exception of measurements in spleen and liver extracts, where the data are expressed as a percentage of 36B4 (housekeeping gene) expression.

**Table 2:** Primer sequences used for quantitative RT-PCR.

Gene Name	Primer Sequence
NOS-2	CTCATGACATCGACCAGAAGCGT
	TATATTGCTGTGGCTCCCATGTTG
COX-2	ATACTGGAAGCCGAGCACCTTTGG
	ATGGTGGCTGTTTTGGTAGGCTGT
Arg-1	AGTCTGGCAGTTGGAAGCATCTCT
	TTCCTTCAGGAGAAAGGACACAGG
Ym-1	CAGGTCTGGCAATTCTTCTG
	GTCTTGCTCATGTGTGTAAGTG
CD206	CCATTTATCATTCCCTCAGCAAGC
	AAATGTCACTGGGGTTCCATCACT
MMP-2	GGCTGACATCATGATCAACTTTGG
	GCCATCAGCCGTTCCCATACTTTAC
MMP-3	GCATCCCCTGATGTCCTCGTGG
	TCCCCGGAGGGTGCTGACTG
MMP-8	TGCCTCGATGTGGAGTGCCTGA
	GCCCTTGACAGCTGTGGCGT
MMP-9	AGAGAGGAGTCTGGGGTCTGGTTT
	GAGAACACCACCGAGCTATCCACT
MMP-12	AATTACACTCCGGACATGAAGCGT
	GGCTAGTGTACCACCTTTGCCATC
MMP-13	ATGATGATGAAACCTGGACAAGCA
	ATAGGGCTGGGTCACACTTCTCTG
MMP-14	ACCACAAGGACTTTGCCTCTGAAG
	CACCGAGCTGTGAGATTCCCTTGA
MMP-19	GATGAACTGGCCAGAACTGACCTT
	GTCCCCGGTTGATGAGTTAGTGTC
MMP-23	CAAGGTTGGTGAGAGAGGGTAGGA
	AGGAGTAGGTGCTGAGAACACGCT
MMP-25	CTCTGAGTGGCAGTGTTTGGAAGA
	TGATGTCAGGCTCCTGGTACTGAG
TIMP-1	AGGAACGAAATTTGCACATCAGT
	CAAAGTGACGGCTCTGGTAGTCCT
TIMP-2	GACTCCCCCTCAGACTCTCCCTAC
	CATATTGATACCACCGCACAGGAA
TIMP-3	CACATCAAGGTGCCATTCAGGTAG
	GTTCTCTCCTCCTCAACCCAAACA
36B4	GCCCAGGGAAGACAGGGCGA
	gcgcatcatggtgttcttgccca
IFNγ	TGCCAAGTTTGAGGTCAACAACCC
	TTTTCCGCTTCCTGAGGGTGGATT
Sort1	TGTGGCCAAGCAGCCATCCG
	TCAGGCTGCTCCACGCACTC
TNFα	CCTGTAGCCCACGTCGTAG
	GGGAGTAGACAAGGTACAACCC
GM-CSF	ATGCCTGTCACGTTGAATGAAGAG
	GGCTGTCTATGAAATCCGCATAGG
IL1β	GCAACTGTTCCTGAACTCAACT
	ATCTTTTGGGGTCCGTCAACT
IL2	TGAGCAGGATGGAGAATTACAGG
	GTCCAAGTTCATCTTCTAGGCAC
IL6	GTTCTCTGGGAAATCGTGGA
	TTCTGCAAGTGCATCATCGT
IL12p40	AGACCAGGCAGCTCGCAGCAAAGCA
	GACACATCCCACTCCCACGCTGCC
IL4	GGTCACAGGAGAAGGGACGCC
	GCGAAGCACCTTGGAAGCCCT
IL5	CTCTGTTGACAAGCAATGAGACG
	TCTTCAGTATGTCTAGCCCCTG
IL10	GCCCCAGGCAGAGAAGCATGGCCC
	ACAGGGGAGAAATCGATGACAGCGCC
Ly6C	GCAGTGCTACGAGTGCTATGG
	ACTGACGGGTCTTTAGTTTCCTT

### Flow cytometry

Peritoneal fluid was collected from EKO and DKO mice immediately after sacrificing the animals; using a lavage of 10 mL of sterile PBS. The cellular components were isolated by centrifugation (1500 rpm, 5 min at 4°C) and red blood cells were lysed using ACK Lysis Buffer (Invitrogen, UK). Remaining cells were resuspended in cold PBS, counted and approximately 1x10^6^ cells/sample assessed for viability, using a 30 min incubation with Viability Dye (eFluor780, 1:1000 diluted in PBS; eBioscience, USA) at 4°C. Cells were subsequently washed with FACS Buffer [25 mM HEPES, 2 mM EDTA, 0.5% v/v FBS in PBS] and non-specific antibody binding sites were blocked by incubation with TrueStain fcX (BioLegend, USA) (containing anti-mouse CD16/32 antibodies mix) for 10 min at 4°C. A freshly prepared mixture of probe-conjugated antibodies (Table 3) was added to each sample. Following 1 h incubation of the primary antibodies (Table 3), cells were washed with FACS buffer and streptavadin-488 to bind to biotinylated F4/80 (1:500 in PBS for 10 min on ice in the dark). Finally, cells were washed once again in FACS buffer and fixed with 1% paraformaldehyde in FACS buffer. To each run, we included a viability dye control (using pooled cells exposed to viability dye only prior to fixation), an unstained control (using pooled cells that were only incubated with TrueStain fcX prior to fixation) and single stained controls for compensation (see next paragraph). We used a BD Biosciences flow cytometer (LSR II) with laser lines set to 405 nm, 488 nm and 633 nm. Optical emission filters used for the corresponding fluorescent reagents were AF488: 530/30 BP, PacBlue: 450/50 BP, AF700: 710/40, PE: 575/26, NIR L/D: 780/60. Data were analysed using the software package FACS Diva 6.1.2. The possibility of instrument-related fluorescence intensity changes over time was excluded by daily run quality control procedures (using CS&T software) and appropriate calibrations.

**Table 3:** Flow Cytometer Set up.

Instrument	BD Biosciences LSR II				
Laser Lines	405	488		633	
Emission Filters	450/50	530/30	575/26	710/40	780/60
Fluorochromes	PacBlue	AF488	PE	AF700	NIR L/D
Antibody/Probe	CD11b-PB	F4/80-Biotin (& streptavadin-488)	CD206-PE	CD11c-AF700	Viability Dye (eFluor780)
Cat.No (Supplier)	Biolegend, 101224	AbD Serotec, UK, MCA497B (& Invitrogen S11223)	Biolegend, 141706	Biolegend, 117320	eBioscience, 65–0865

Multi-colour compensation was performed by using OneComp eBeads (eBioscience, 01–1111) for single-colour controls. We analysed a minimum of 10000 events/sample. Statistical analysis used the percentage proportion(s) of cells within specific gates rather than fluorescence intensities. The gating tree was set as follows: A) FCS/SSC—representing distribution of cells based on size and intracellular composition, respectively, to B) live gate (eFluor780 negative cells)–representing the fraction of viable cells to C) F480/CD11b double positive cells–representing mature macrophages to D) stained with CD11c or CD206 (M1/M2 markers, respectively).

### Histological and immunohistochemical methods

The BCA from each mouse was supported in agar and embedded in paraffin. Sections were cut at 3 μm intervals from its origin at the aorta, and stained and analysed as described previously [34, 35]. Briefly, the first section after the bifurcation of the BCA from the aorta was cleared and rehydrated and then stained using Miller’s elastin/van Gieson (EVG) and plaque dimensions were measured using image analysis software (Image Pro, DataCell, UK). The AS from each mouse was treated and examined in a similar fashion.

For immunohistochemistry (IHC), 3 μm paraffin sections on glass slides were de-waxed and rehydrated. Endogenous peroxidase activity was blocked by incubating the slides in 3% H_2_O_2_ in PBS. Antigen retrieval was performed using 10 mM citrate buffer pH 6.0 (2x5 min in a microwave oven). Non-specific epitopes were blocked with 10% goat serum in 1% BSA:PBS and the sections were subsequently incubated with primary antibodies for smooth muscle cells (α-smooth muscle actin; SMA), macrophages (*Griffonia simplicifolia* lectin (GSL) I), NOS-2, COX-2, CD206, Arg-1, Ym-1, MMP-12, MMP-13, MMP-14 and TIMP-3 (Table 4A) overnight at 4°C. Negative controls were included in every run, where IgG from the appropriate host animal substituted the primary antibody. Sections were then washed and incubated with a biotinylated secondary antibody (Table 4B) followed by Extravidin-HRP (Sigma) and DAB (Sigma). Nuclei were counterstained using haematoxylin (Sigma). The areas stained with each cell-specific or phenotypic marker or MMP/TIMPs antibody were determined using the same image analysis software detailed above and reported as percentage of the total plaque area. The number of buried layers was assessed manually on sections stained for elastin (EVG) and confirmed on sections stained for SMA.

**Table 4:** Primary (A) and secondary (B) antibodies and Griffonia simplicifolia Lectin I (GSL).

**A**	**Primary Antibody**	**Supplier/Cat. No**	**Type**	**Dilution Used (IHC)/ Final Concentration**
	GSL I	VectorLabs, USA B1215	Lectin	1:100
	SMA	Dako, M0851	Mm_Mab	1:100
	NOS-2	Abcam, UK #ab15323	Rb_PAb	1:75
	COX-2	Abcam #ab15191	Rb_PAb	1:100
	Arginase-1	Santa Cruz Biotechnology, USA sc-20150	Rb_PAb	1:100
	Ym-1/2	StemCell Technologies, Canada #01404	Rb_PAb	1:100
	MMP-12	Abcam #ab52897	Rb_MAb	1:50
	MMP-13	Abcam #ab39012	Rb_PAb	1:100
	MMP-14	Abcam #ab51074	Rb_MAb	1:50
	TIMP-3	Abcam #ab39206	Rb_PAb	1:1000
	BrdU	Sigma, B2531	Mm_Mab	1:500
	Rb IgG1	Sigma, I5006	Neg. Control	As needed
	Mm IgG2a	Sigma, M5409	Neg. Control	As needed
**B**	**Secondary Antibody**	**Supplier/Cat. No**	**Type**	**Dilution Used (IHC)/ Final Concentration**
	Gt^Mm	Dako, E0433	biotinylated	1:300
	Gt^Rb	Dako, E0432	biotinylated	1:300

Aortae from formalin-fixed animals were carefully cleaned of adherent fat and opened longitudinally *in situ*. Lipid-rich areas in these vessels were stained with 2% Oil-Red-O (ORO, Sigma) in isopropanol and digitalised *en face*. The area occupied by ORO-stained lesions was determined using NIH ImageJ v1.43 and is expressed as a proportion of the total vessel area.

### Statistical methods

All analyses were performed using GraphPad InStat v3.05 (GraphPad Software Inc, USA) or SPSS v21 (IBM, USA) software. Data were checked for normality (Kolmogorov and Smirnov normality test). Regression analyses were performed using Pearson’s correlation co-efficient. Statistical analyses of data were performed using Students *t*-test, or 1- or 2-way ANOVAs, with the 1-way ANOVA followed by a Bonferroni or Tukey-Kramer post-test. The Mann-Whitney U-test or logarithmic transformation was used for non-normally distributed data. Normally distributed data are expressed as arithmetic mean ± SEM. Statistical significance is defined as P<0.05.

## Results

### Expression of MMPs and TIMPs in mouse macrophages during classical activation in vitro

**Fig 1:**
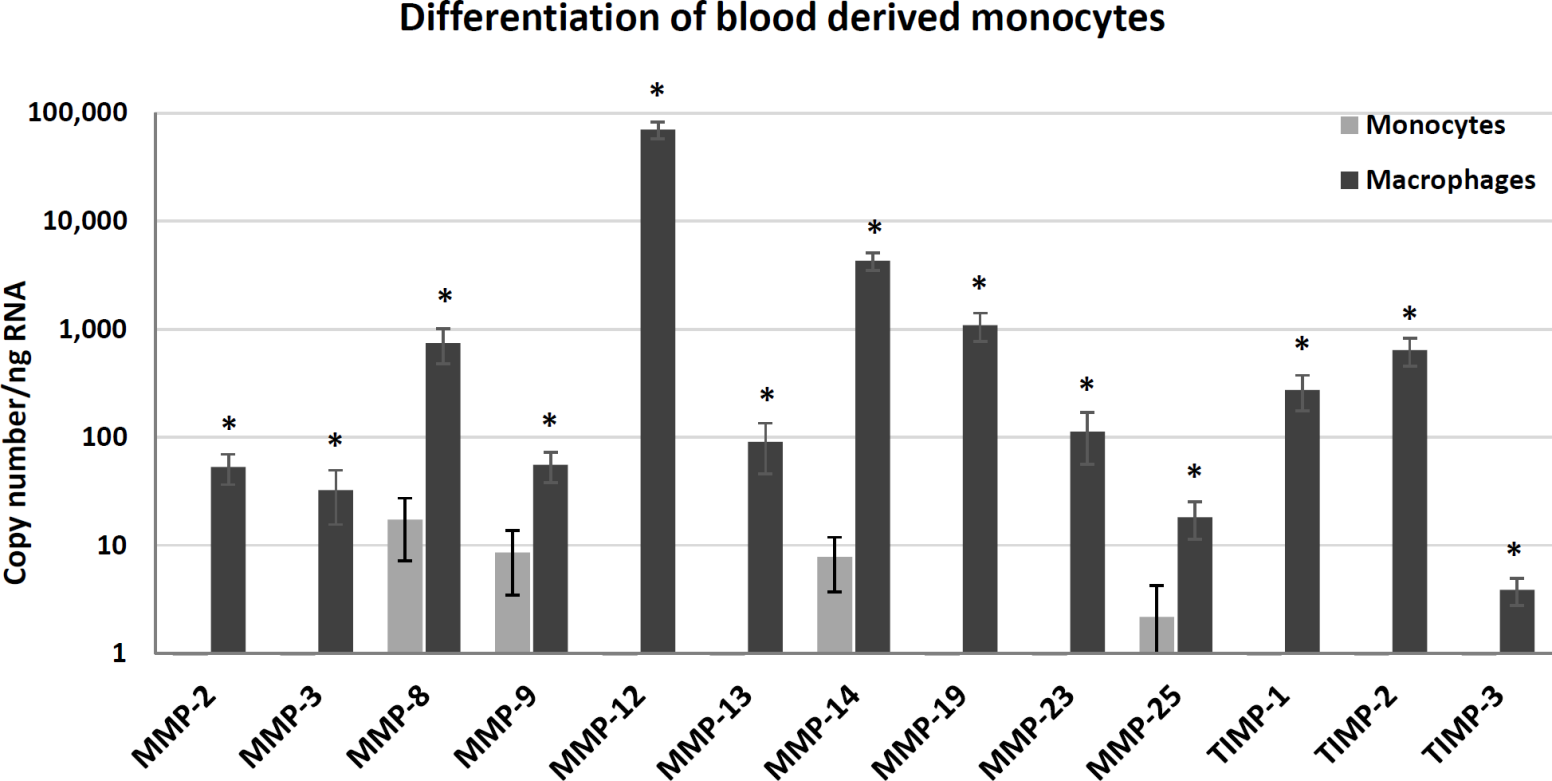
Effects on MMP and TIMP mRNA levels of M-CSF-driven macrophage maturation in vitro. MMP and TIMP mRNA levels in blood monocytes and MCSF-differentiated macrophages *in vitro*. Steady-state mRNA levels were measured by RT-qPCR in undifferentiated, non-adherent, CD11b positively selected blood monocytes (Monocytes) or monocyte-derived macrophages differentiated *in vitro* in M-CSF for 7 days (Macrophages). Values are expressed as mean ± SEM. * P<0.05 (N = 3).

To investigate which MMPs and TIMPs are abundantly expressed and may be increased by classical (M1) activation we conducted experiments on macrophages differentiated from blood monocytes with M-CSF. Non-adherent blood monocytes only expressed detectable mRNA levels for MMP-8>MMP-9 = MMP-14>MMP-25 (Fig 1). Remarkably, many MMPs were strongly and significantly induced during differentiation of monocytes to macrophages using M-CSF (Fig 1). Transcript copy numbers varied widely with MMP-8, MMP-12, MMP-14, MMP-19, TIMP-1 and TIMP-2 being especially abundant (Fig 1). MCSF-differentiated macrophages were then treated with IFNγ, TNFα or the combination to investigate the influence of M1 activation on MMPs and TIMPs. None of the MMP transcripts was significantly induced by IFNγ (Table 5). MMP-2, MMP-9, and MMP-14 transcripts were induced to a similar level by TNFα in the presence or absence of IFNγ (Table 5). The mRNA levels of TIMP-2 were suppressed by TNFα, whereas those of other MMPs and TIMPs were unaffected (Table 5). These data suggest that a relatively small subgroup of MMPs and TIMPs is regulated by classical macrophage activation in mouse blood monocyte-derived macrophages *in vitro*.

**Table 5:** Comparison of MMP and TIMP mRNA levels after treatment with IFNγ and TNFα compared with untreated controls (copy number per ng RNA).

Treatment	Control (N = 3)		IFNγ (N = 3)			TNFα (N = 3)			IFNγ + TNFα (N = 3)		
Gene	Mean	SEM	Mean	SEM	*P value*	Mean	SEM	*P value*	Mean	SEM	*P value*
MMP-2	53	16	32	20	*0*.*375*	195	58	***0*.*014***	173	15	***0*.*050***
MMP-3	33	17	49	37	*0*.*908*	59	34	*0*.*638*	25	8	*0*.*929*
MMP-8	744	268	733	191	*0*.*746*	737	180	*0*.*771*	427	109	*0*.*491*
MMP-9	56	18	48	19	*0*.*632*	404	66	***0*.*016***	506	70	***0*.*019***
MMP-12	70097	12719	74065	5569	*0*.*713*	105010	16959	*0*.*364*	67815	25118	*0*.*719*
MMP-13	90	44	52	14	*0*.*286*	12	1	*0*.*108*	5	2	*0*.*074*
MMP-14	4286	772	4175	1459	*0*.*773*	73506	4461	***0*.*006***	79766	3543	***0*.*004***
MMP-19	1089	320	1166	115	*0*.*537*	1216	169	*0*.*504*	591	108	*0*.*238*
MMP-23	113	57	120	57	*0*.*920*	43	19	*0*.*422*	37	17	*0*.*172*
MMP-25	18	7	47	5	*0*.*149*	106	20	*0*.*079*	72	18	*0*.*090*
TIMP-1	275	99	279	167	*0*.*861*	97	62	*0*.*103*	137	55	*0*.*085*
TIMP-2	639	184	563	75	*0*.*946*	324	79	***0*.*033***	411	20	*0*.*407*
TIMP-3	4	1	2	2	*0*.*317*	0	0	*0*.*070*	1	0	*0*.*064*

### Characterization of ApoE knockout (EKO) and ApoE/T-bet double knockout (DKO) mice fed a HFD

To investigate the impact of preventing differentiation of Th1 lymphocytes on plaque development, M1/M2 macrophage polarization and MMP and TIMP expression in FCMs we compared homozygous EKO and ApoE/T-bet double knockout (DKO) mice.

### Lipid profiles

To induce atherosclerosis rapidly, we fed the EKO and DKO mice with HFD for 12 weeks. We then measured the levels of cholesterol-rich lipoproteins in the plasma. The plasma concentrations of total cholesterol and LDL+VLDL were not different between EKO and DKO mice. However, HDL cholesterol was increased 2.4-fold in DKO mice (Table 6). Based on the increase in HDL, DKO mice might be protected from atherosclerosis compared with EKO mice.

**Table 6:** Concentration of cholesterol-containing lipids in plasma from HFD EKO and DKO mice.

Cholesterol Lipids	EKO 12 wk HFD (N = 7)		DKO 12 wk HFD (N = 7)		
[mg/dL]	Mean	SEM	Mean	SEM	*P value*
TOTAL	1159.1	15.3	1126.9	76.1	*0*.*383*
HDL	52.6	8.5	128.2	28.1	***0*.*007***
LDL & VLDL	1106.5	19.3	998.7	93.9	*0*.*717*

### Cytokine profiles and splenic levels of Ly6C

The T-bet transcription factor stimulates IFNγ expression and is also induced by IFNγ, a positive feedback loop that leads to increased IFNγ production and Th1 lymphocyte differentiation [26]. Nevertheless, we found no difference in mRNA expression of IFNγ in the livers or spleens of HFD EKO compared to DKO mice (Table 7), which demonstrates that T-bet independent sources of IFNγ predominate under these conditions. Furthermore, when we measured the expression of a panel of other cytokines related to Th1 and Th2 lymphocytes, none showed a significant change in the spleen. In addition, we used a commercially-available multiplex panel for the same cytokines to measure blood concentrations. Levels of IL-2, IL-4 and IL-5 were too low and variable to be informative in any set of mice but the other 5 cytokines gave consistent values (Table 7). IL-10, IL12p70, GM-CSF, IFNγ and TNFα showed no differences between genotypes after 12 weeks of HFD, consistent with the splenic mRNA data.

**Table 7:** mRNA levels and plasma cytokine concentrations in HFD EKO and DKO mice.

	Spleen (liver) mRNA [% of 36B4)					Plasma cytokine [pg/mL]				
Cytokine	EKO 12wk HFD (N = 8)		DKO 12wk HFD (N = 8)			EKO 12wk HFD (N = 9)		DKO 12wk HFD (N = 10)		
	Mean	SEM	Mean	SEM	*P value*	Mean	SEM	Mean	SEM	*P value*
IFNγ (liver)	2.894	0.747	3.674	0.254	*0*.*710*					
IFNγ	1.006	0.282	0.619	0.371	*0*.*232*	10	2.9	15.7	9.2	*0*.*910*
TNFα	0.240	0.148	0.138	0.056	*0*.*517*	249.3	55.8	448.7	174.5	*0*.*549*
GM-CSF	2.058	1.521	1.807	1.768	*0*.*447*	124.7	36.6	106.1	24	*0*.*905*
IL12p40	5.105	3.763	5.657	4.720	*0*.*161*	92	31.9	74.1	18.8	*0*.*968*
IL2	0.0052	0.0052	0.0000	0.0000	*0*.*242*	44.5	17.4	24.3	13.9	*0*.*447*
IL4	0.039	0.009	0.033	0.009	*0*.*574*	3.3	1.3	21.7	12.8	*0*.*604*
**IL5**	**0.025**	**0.011**	**0.063**	**0.015**	***0*.*036***	12.5	5.2	76.4	44.1	*0*.*549*
IL10	0.076	0.027	0.052	0.036	*0*.*394*	33.8	4.7	23.1	4.4	*0*.*211*
Ly6C	1.232	1.005	0.312	0.263	*0*.*315*					* *

Previous research shows that HFD increases the levels of Ly6C plus monocytes in spleens and their mobilization into the circulation where they provide a source of activated plaque macrophages [36]. There were, however, no differences between DKO and EKO mice and hence the effect on Ly6C mRNA levels was independent of T-bet.

### Activation status of peritoneal macrophages

**Fig 2:**
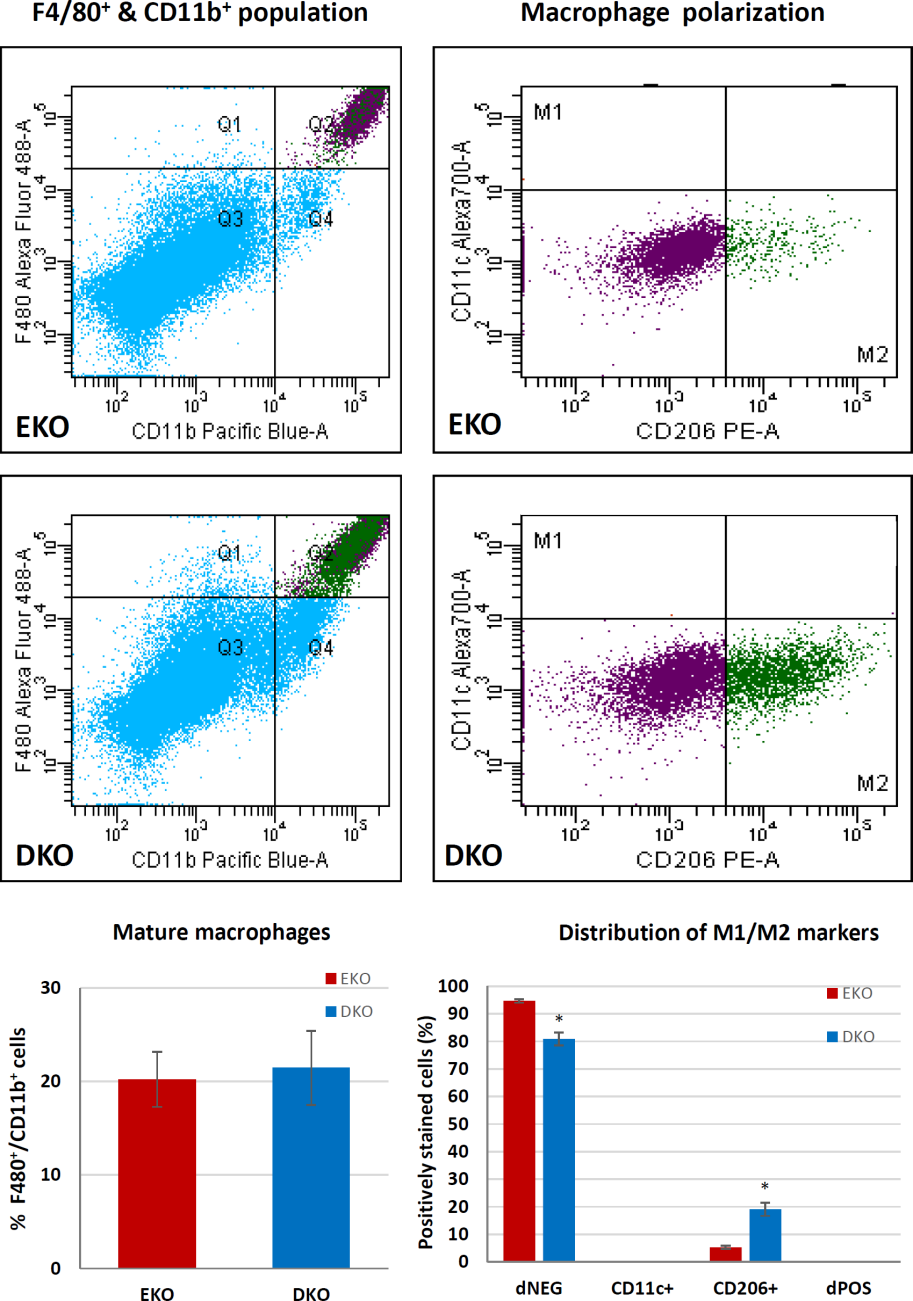
Flow cytometry of peritoneal fluid macrophages. Flow cytometry of peritoneal fluid macrophages. Viable mature macrophages (F4/80^+^ and CD11b^+^) from peritoneal lavage fluid were subsequently categorized according to their expression of M1 (CD11c+) and/or M2 (CD206) markers. Values are expressed as mean ± SEM. * P<0.05 (N = 9–10).

To further characterize the effects of T-bet knockout, resident peritoneal macrophages were collected from EKO and DKO mice fed a HFD for 12 weeks and prepared for flow cytometry, using antibodies to identify mature macrophages (F4/80 and CD11b) as well as markers of classical (CD11c) and alternative (CD206) macrophage activation, characterised previously [37]. Mature macrophages positive for both F480 and CD11b were seen in all samples from both genotypes, comprising approximately 20% of all the cells collected (Fig 2). M1 macrophages (or indeed any other cell types) positive for CD11c were not detected in either genotype but the percentage of CD206-positive (M2) macrophages was significantly higher in DKO (19%) than EKO (5%) mice (Fig 2). The remaining macrophages were negative for both CD11c and CD206. This confirms previous work indicating that peritoneal macrophages have low levels of M1 and higher levels of M2 polarization [38, 39]. Moreover, our data indicates that knocking out T-bet biases macrophage responses towards M2, as expected [28].

### Phenotypes of FCMs obtained from subcutaneous granulomas in EKO and DKO mice fed a HFD

We first investigated the influence of deleting T-bet on the levels of M1 and M2 markers, MMPs and TIMPs in granuloma FCMs from subcutaneous sponges placed into mice fed a HFD for 4 weeks. The yield of FCMs purified by flotation (i.e. buoyant density <1) was 2.7 ± 0.41 and 3.0 ± 0.71 x 10^6^ cells per EKO and DKO mouse, respectively. These FCMs abundantly expressed both M1 (NOS-2, COX-2 and IL-6) and M2 (CD206, Arg-1, FIZZ1, IL-10 and Ym-1) macrophage markers (Table 8), consistent with previous work [16, 31, 33]. Amongst the M1 markers, NOS-2 was decreased significantly in DKO vs EKO mice and COX-2 and IL-6 showed the same trend (Table 8), which provides evidence for polarization away from M1 in the absence of Th1 cells. Expression of the M2 markers, Arg-1, CD206, FIZZ1, IL-10 and Ym-1 was similar in DKO vs EKO mice. Consistent with the effect on NOS-2, TIMP-2 levels, which were decreased by M1 conditions *in vitro*, were increased in DKO vs EKO mice. However, of the MMPs that were stimulated by M1 activation *in vitro*, MMP-2 was paradoxically increased in EKO vs DKO mice and MMP-14 showed the same trend, whereas MMP-9 showed no difference. Furthermore, pilot experiments using *in situ* zymography found no difference in gelatinase activity between foam cells of EKO and DKO mice (results not shown). Hence, although preventing Th1 cell differentiation reduced M1 polarization in granuloma FCMs, the levels of MMP mRNAs were not changed or actually increased. FCMs isolated from subcutaneous granulomas have measureable rates of proliferation [31, 33]. Immunocytochemistry after an 8 h BrDU pulse showed a significantly increased proliferation rate in DKO compared to EKO mice (11.45±1.51% vs 6.96±0.74%, N = 8–11, P<0.05).

**Table 8:** Levels of mRNA transcripts (copies per ng of total RNA) in granuloma macrophages.

Gene	EKO 12 wk HFD (N = 8)		DKO 12 wk HFD (N = 6)		* *
Name	Mean	SEM	Mean	SEM	*P value*
NOS-2	69	10	33	11	***0*.*034***
COX-2	762	78	576	117	*0*.*193*
IL-6	56	8	43	7	*0*.*254*
Arg-1	52971	7061	44141	8684	*0*.*441*
Ym-1	9	3	11	4	*0*.*618*
CD206	2197	521	2227	378	*0*.*965*
FIZZ1	440	128	613	164	*0*.*413*
IL-10	3539	567	4334	649	*0*.*376*
MMP-2	243	34	435	56	***0*.*009***
MMP-9	106	17	99	17	*0*.*776*
MMP-12	67164	7863	74200	9786	*0*.*581*
MMP-13	12836	2273	9767	2197	*0*.*363*
MMP-14	2335	301	2905	179	*0*.*162*
TIMP-1	1245	168	1529	277	*0*.*374*
TIMP-2	6277	605	9213	1321	***0*.*048***
TIMP-3	1416	182	1510	177	*0*.*724*

### Transcript levels of M1 and M2 markers, MMPs and TIMPs in whole aortae from EKO and DKO mice fed a HFD for 12 weeks

**Fig 3:**
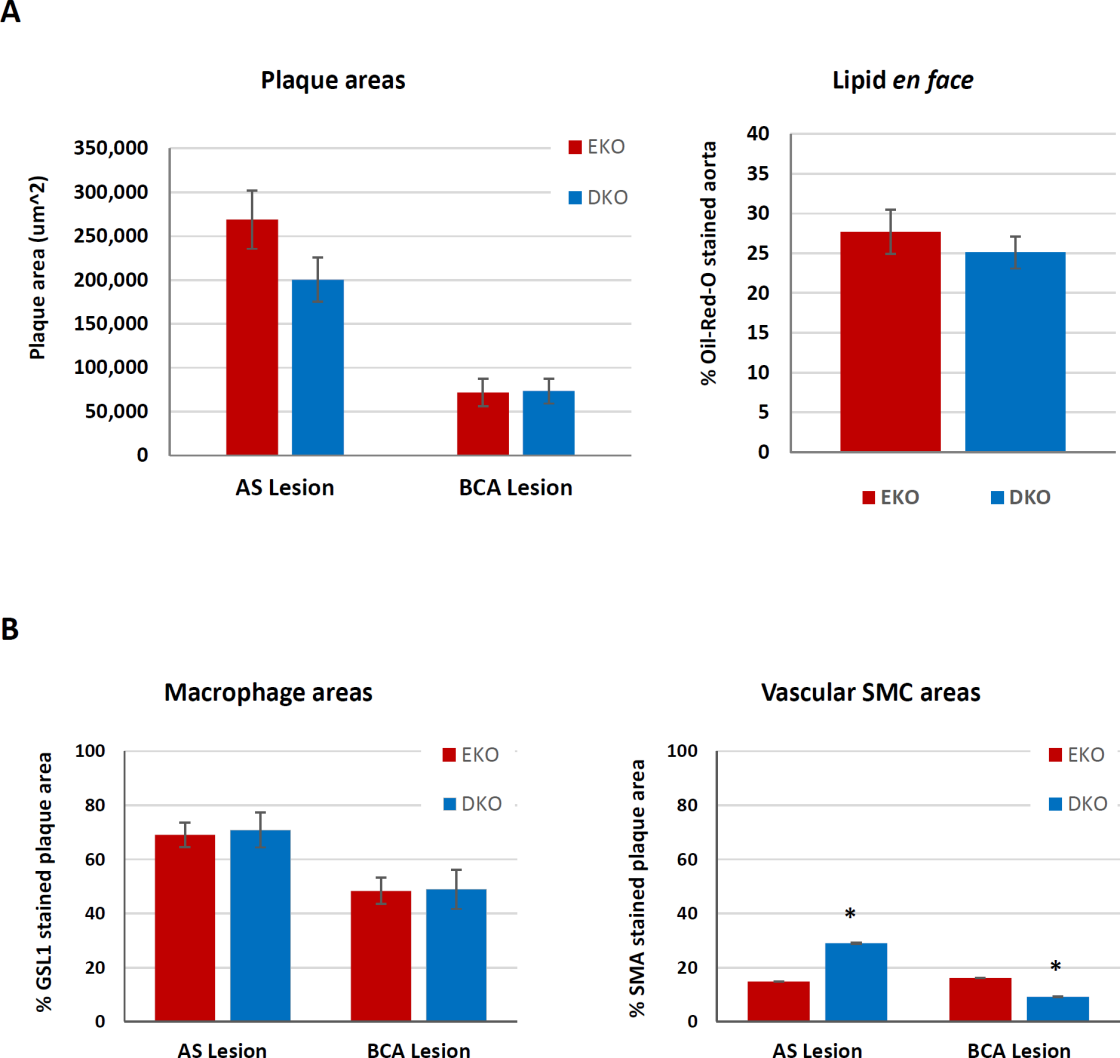
Atherosclerotic plaque size and composition after 12 weeks of HFD in EKO vs DKO mice. Plaque size and composition in EKO vs DKO mice fed a HFD for 12 weeks. (A) AS and BCA plaques were taken from EKO (N = 15) and DKO (N = 10–11) mice. A) Average plaque areas were quantified from the 12 near serial sections subjected to EVG or other cell-specific or other stains. (B) Oil-red-O staining was used to stain for lipids *en face* and the percentage area of lipids in whole aortae was quantified (N = 10). Further sections of AS and BCA plaque were stained for the presence of (C) macrophages (GSL I) and (D) smooth muscle cells (SMA). Values are expressed as mean ± SEM. * P<0.05.

Quantitative RT-qPCR of mRNAs extracted from whole aortae from mice fed a HFD for 12 weeks did not reveal any significant differences in M1 or M2 markers, MMPs or TIMP mRNAs between EKO and DKO mice fed a HFD for 12 weeks (Table 9). This was consistent with the generation of similar sized atherosclerotic plaques in these aortas (Fig 3).

**Table 9:** Levels of mRNA transcripts (copies per ng of total RNA) in extracts of mouse aortae.

Gene	EKO 12 wk HFD (N = 9)		DKO 12 wk HFD (N = 8)		* *
Name	Mean	SEM	Mean	SEM	*P value*
NOS-2	33	12	43	17	*0*.*6318*
COX-2	698	159	696	139	*0*.*9927*
Arg-1	7	6	8	4	*0*.*8944*
Ym1	6	2	8	3	*0*.*5795*
MMP-2	650	112	559	114	*0*.*5785*
MMP-9	44	7	48	6	*0*.*6746*
MMP-12	1937	566	1792	538	*0*.*8562*
MMP-13	473	141	377	112	*0*.*6081*
MMP-14	3110	447	3107	651	*0*.*9970*
MMP-19	410	107	840	228	*0*.*0965*
TIMP-1	1123	374	1798	730	*0*.*4078*
TIMP-2	8504	1022	8498	876	*0*.*9965*
TIMP-3	30193	5493	27460	3997	*0*.*6996*

### Atherosclerotic plaque size and composition in EKO vs DKO mice fed a HFD for 12 weeks

Atherosclerotic lesions develop rapidly after HFD in the aortic sinus (AS) and the aortic arch [40], especially at the origin of the brachiocephalic artery (BCA) in EKO mice [41]. From histological sections, plaques in the AS and BCA were not different in DKO and EKO mice (Fig 3A), although there was a trend to smaller plaques in the AS of DKO mice. We also looked at lesion formation *en face* using the whole aorta and found no difference between EKO and DKO mice (Fig 3B). Furthermore, we counted the number of buried fibrous layers in BCA plaques, as a marker of plaque complexity or instability [42]. We found no significant differences between genotypes, with EKO having 1.5 ± 0.2 buried layers per plaque and DKO having 1.6 ± 0.2 buried layers per plaque (N = 9–11, P = 0.9749).

**Fig 4:**
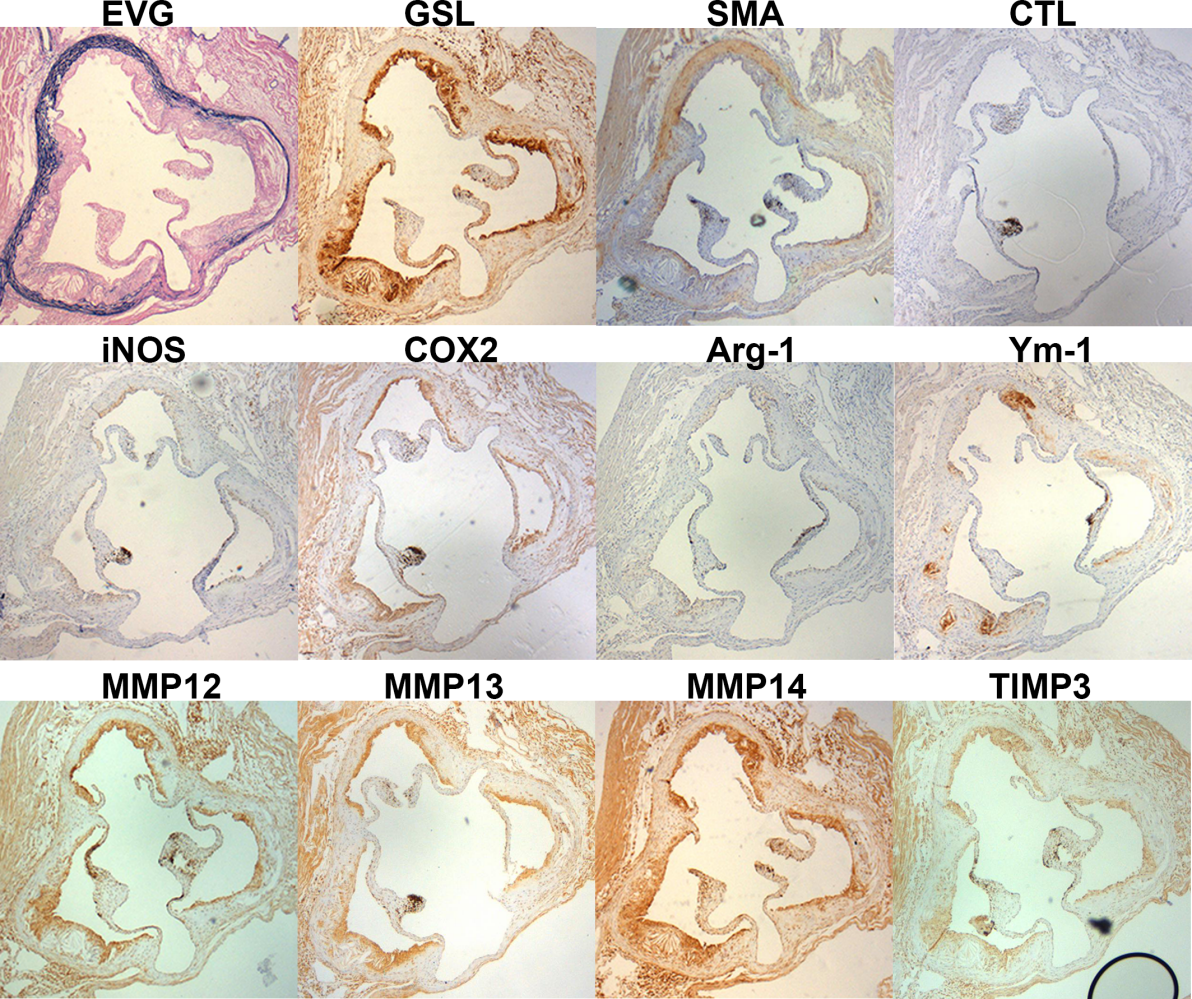
Immunohistological staining for cell type, M1 and M2 markers, MMPs-12, -13 and -14 and TIMP-3 after 12 weeks of HFD. Immunohistochemical staining for cell type, M1/M2 markers, selected MMPs and TIMP-3 after 12 weeks of HFD. Near consecutive sections to the sections stained with EVG were stained for macrophages (GSL I), smooth muscle cells (SMA), M1 markers (NOS-2 & COX-2), M2 markers (Arg-1 and Ym-1), selected MMPs-12, -13 and -14 or TIMP-3, as indicated. Isotype controls were performed with non-immune IgG replacing the primary antibody in each run. This figure shows stained sections of AS plaques from an EKO mouse as a representative example.

*Griffonia simplicifolia* Lectin I (GSL) was used to stain plaque macrophages (Fig 4), the majority of which appeared to be FCMs. Some medial cells also stained with GSL, previously identified as foamy vascular SMCs transdifferentiating towards macrophages [43]. However, the media was not included in our analyses. We found no differences in the percentage of plaque area stained for GSL at either site between DKO and EKO mice (Fig 3C). Interestingly, AS plaques were significantly more macrophage-rich than BCA plaques (approximately 60% vs 45%, respectively). IHC for smooth muscle α-actin (SMA) was used to quantify the presence of SMCs, which were mainly located in the fibrous cap of plaques, as expected (Fig 4). EKO mice had approximately 15% of their plaques stained for SMA in either site (Fig 3), consistent with the expected lipid-rich nature of plaques in this model at this time point. DKO mice had approximately 2 fold more SMA in their AS plaques and approximately 2 fold less SMA in their BCA plaques than EKO mice (Fig 3).

### M1 and M2 markers, MMP-12, -13 and -14 and TIMP-3 protein expression in atherosclerotic plaques of EKO vs DKO mice after 12 weeks of HFD

We used IHC to observe and quantify the protein expression of the M1 markers, NOS-2 and COX-2, and M2 markers, Arg-1 and Ym-1 (Fig 4). We also studied the protein expression of MMP-12, MMP-13 and MMP-14 and TIMP-3 because of their prominent roles in plaque growth and stability (see Discussion). The staining was clear and specific (Fig 4) and we measured the percentage of total plaque that stained with each antibody. Although some of the antibodies stained cardiac myocytes surrounding the aortic root, this did not affect our quantification because only areas within plaques were measured.

**Fig 5:**
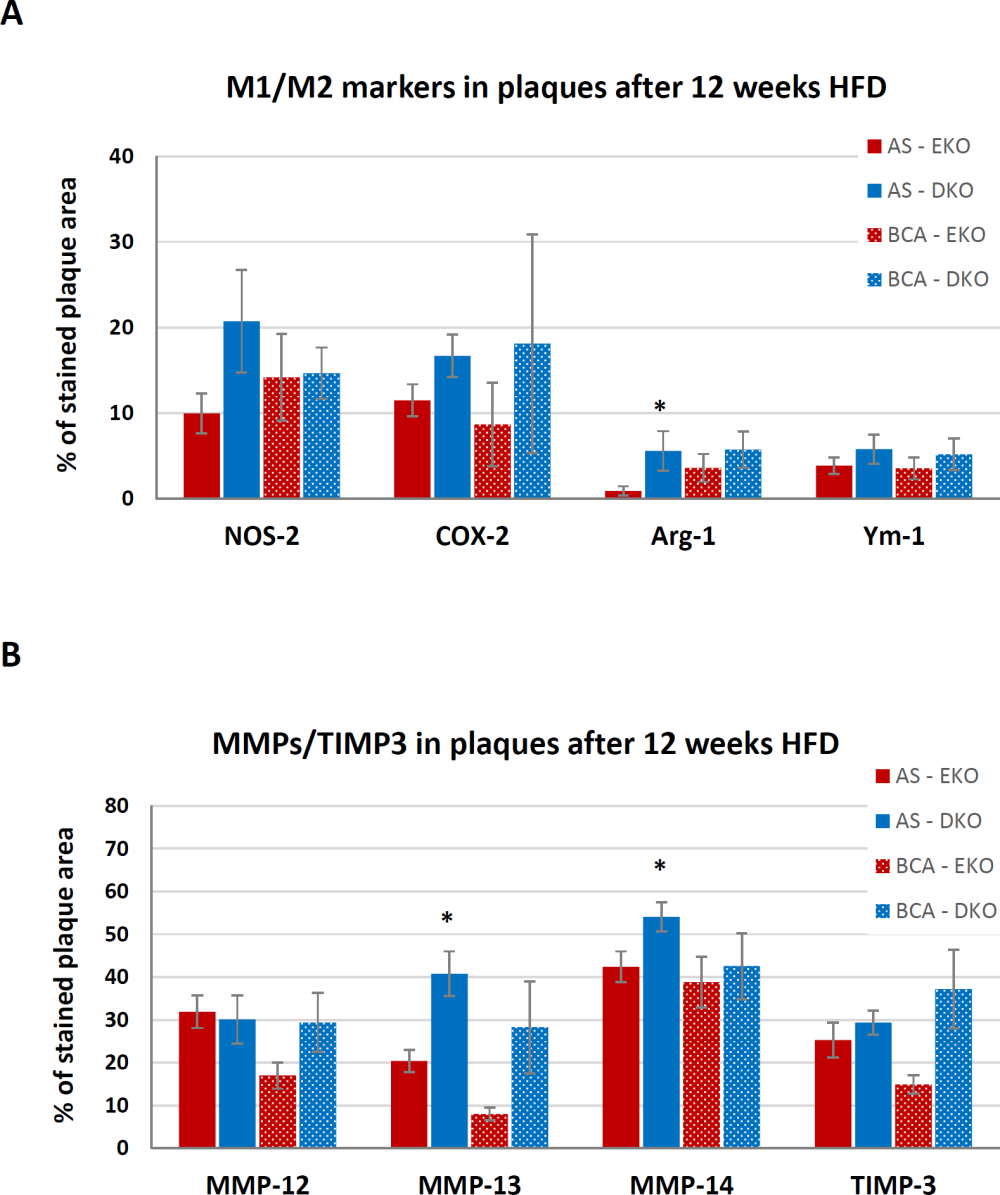
Expression of M1/M2 markers and selected MMPs/TIMP-3 in plaques of HFD EKO and DKO mice. Quantification of M1 and M2 markers, MMPs and TIMP-3 in atherosclerotic plaques of EKO (N = 9–15) and DKO (N = 5–11) mice after 12 weeks of HFD. Near consecutive sections were subjected to IHC for (A) NOS-2 and COX-2 (as M1 markers), Arg-1 and Ym-1 (as M2 markers) and (B) MMPs-12, -13, -14 and TIMP-3. Values are expressed as mean ± SEM. * P<0.05.

After 12 weeks of HFD, there was no significant difference in the areas stained for either NOS-2 or COX-2 in AS or BCA plaques of DKO compared to EKO mice (Fig 5A). Hence it appears that Th1 lymphocytes are not required for M1 polarization in atherosclerotic plaque FCMs after 12 weeks of HFD. AS plaques of DKO mice had a significantly increased percentage area positive for Arg-1 when compared to EKO mice (5.6±2.3 vs 0.9±0.5%, N = 7–10), suggesting increased polarization towards M2, but this was not observed in the BCA (Fig 5A). Moreover, we observed no significant differences in the percentage of BCA plaque area stained for Ym-1 between DKO and EKO mice (Fig 5A). Hence there was only weak evidence for increased M2 polarization of plaque FCMs in the absence of Th1 cells under these conditions. Staining for MMP-13, which increases on M1 activation, was paradoxically increased by approximately 2 fold in DKO vs EKO mice at both sites, although the increase was statistically significant only in AS plaques (P = 0.0090) (Fig 5B). Staining for MMP-14, which is also M1 related, was similarly increased in the AS plaques of DKO vs EKO mice (where P = 0.032). There were no significant differences between genotypes in MMP-12 or TIMP-3 staining at either site studied.

### Comparison of plaques from EKO vs DKO mice fed a normal diet (ND) for 35 weeks

It was previously shown that deletion of T and B cells had no effect on lesion size in EKO mice fed a HFD [44, 45] but resulted in a reduced lesion size in mice fed a ND for a longer period [44, 46]. This led to the concept that the greater inflammatory effect of the HFD could overwhelm the protective effect of deleting adaptive immunity [47]. To allow for this possible confounding effect in our experiments, we also studied EKO and DKO mice fed a ND, under which conditions plaque development is known to be considerably slower [40]. Following pilot observations showing small and highly variable plaques in mice fed a ND until 30 weeks of age (data not shown), we fed EKO and DKO male mice a ND until 35 weeks of age.

### Lipid profiles

Terminal plasma LDL plus VLDL concentrations were significantly lower after 35 weeks of ND compared to 12 weeks of HFD (19%, P = 0.0003 for EKO and 27%, P = 0.0012 for DKO) (Table 10 compared to Table 6), consistent with previous more extensive observations [40, 48]. However, concentrations of total cholesterol or LDL plus VLDL were not different between EKO and DKO mice after 35 weeks of ND (Table 10). HDL cholesterol was significantly increased in DKO compared to EKO mice (by 1.6 fold), as it was in mice fed a HFD (Table 6). Based on the increase in HDL, DKO mice might be protected from atherosclerosis compared with EKO mice.

**Table 10:** Concentration of cholesterol-containing lipids in plasma from ND EKO and DKO mice.

Cholesterol Lipids	EKO 35 wk ND (N = 7)		DKO 35 wk ND (N = 7)		
[mg/dL]	Mean	SEM	Mean	SEM	*P value*
TOTAL	935.9	73.0	794.5	92.8	*0*.*270*
HDL	41.9	6.3	65.2	6.5	***0*.*038***
LDL & VLDL	894.1	75.4	729.3	90.2	*0*.*218*

### Cytokine profiles and splenic levels of Ly6C

Consistent with the expected effect of T-bet knockout, we observed a significant reduction in liver and spleen IFNγ mRNA levels in DKO compared to EKO mice fed a ND (Table 11). Hence, under ND, T-bet dependent sources of IFNγ must predominate. Despite this, the levels of all the other cytokine mRNAs measured were not different, except IL-2 which was increased in DKO compared with EKO mice. By contrast to the effects in the spleen and liver, circulating levels of IFNγ were 20-fold higher in DKO compared to EKO mice, demonstrating that T-bet independent sources of IFNγ predominate in this compartment. Levels of IL-12p70, GM-CSF and TNFα, were also approximately 3–20 fold higher in DKO compared with EKO mice (Table 11). Levels of IL-10, which is associated with Th2 lymphocytes and alternative macrophage activation, were also increased approximately 4-fold in DKO compared to EKO mice.

**Table 11:** mRNA levels and plasma cytokine concentrations in ND EKO and DKO mice.

	Spleen (liver) mRNA [% of 36B4)					Plasma cytokine [pg/mL]				
Cytokine	EKO 35wk ND (N = 8)		DKO 35wk ND (N = 8)			EKO 35wk ND (N = 10)		DKO 35wk ND (N = 10)		* *
	Mean	SEM	Mean	SEM	*P value*	Mean	SEM	Mean	SEM	*P value*
**IFNγ (liver)**	**7.075**	**0.760**	**9.176**	**1.881**	***0*.*024***					* *
**IFNγ**	**0.230**	**0.102**	**0.045**	**0.013**	***0*.*004***	**0.4**	**0.3**	**7.6**	**2.6**	***0*.*005***
TNFα	0.667	0.150	0.587	0.221	*0*.*645*	95.6	10.5	245.5	51.7	*0*.*018*
**GM-CSF**	0.122	0.094	0.035	0.019	*0*.*642*	**21.1**	**6.6**	**115.6**	**29.9**	***0*.*002***
IL12p40	0.068	0.026	0.292	0.270	*0*.*442*	20.3	3.4	221.3	84.9	*0*.*000*
**IL2**	**0.0145**	**0.0076**	**0.0549**	**0.0166**	***0*.*028***	3.5	0.0	15.1	8.1	*0*.*481*
IL4	0.055	0.008	0.035	0.010	*0*.*141*	1.5	1.0	7.1	2.9	*0*.*123*
IL5	0.024	0.011	0.046	0.032	*0*.*696*	7.4	3.2	34.5	14.7	*0*.*218*
**IL10**	0.059	0.015	0.041	0.012	*0*.*302*	**8.0**	**2.3**	**29.8**	**9.8**	***0*.*009***
Ly6C	6.309	1.649	4.773	1.607	*0*.*442*					

Only the splenic mRNA levels of IL-12p40 were greater between 12 weeks of HFD and 35 weeks of ND (Table 11 compared to Table 7). However, blood levels of IFNγ, TNFα, GM-CSF, IL-12p70 and IL-10 were significantly lower in EKO mice fed a ND for 35 weeks compared with those fed a HFD for 12 weeks (all P<0.05) (Table 11 compared with Table 7). This is consistent with previous studies at 12 weeks for both diets, which demonstrates the pro-inflammatory effect of HFD [49].

Splenic levels of Ly6C mRNA were not different on EKO and DKO mice under ND, similar to the results from HFD.

### Plaque size and composition

**Fig 6:**
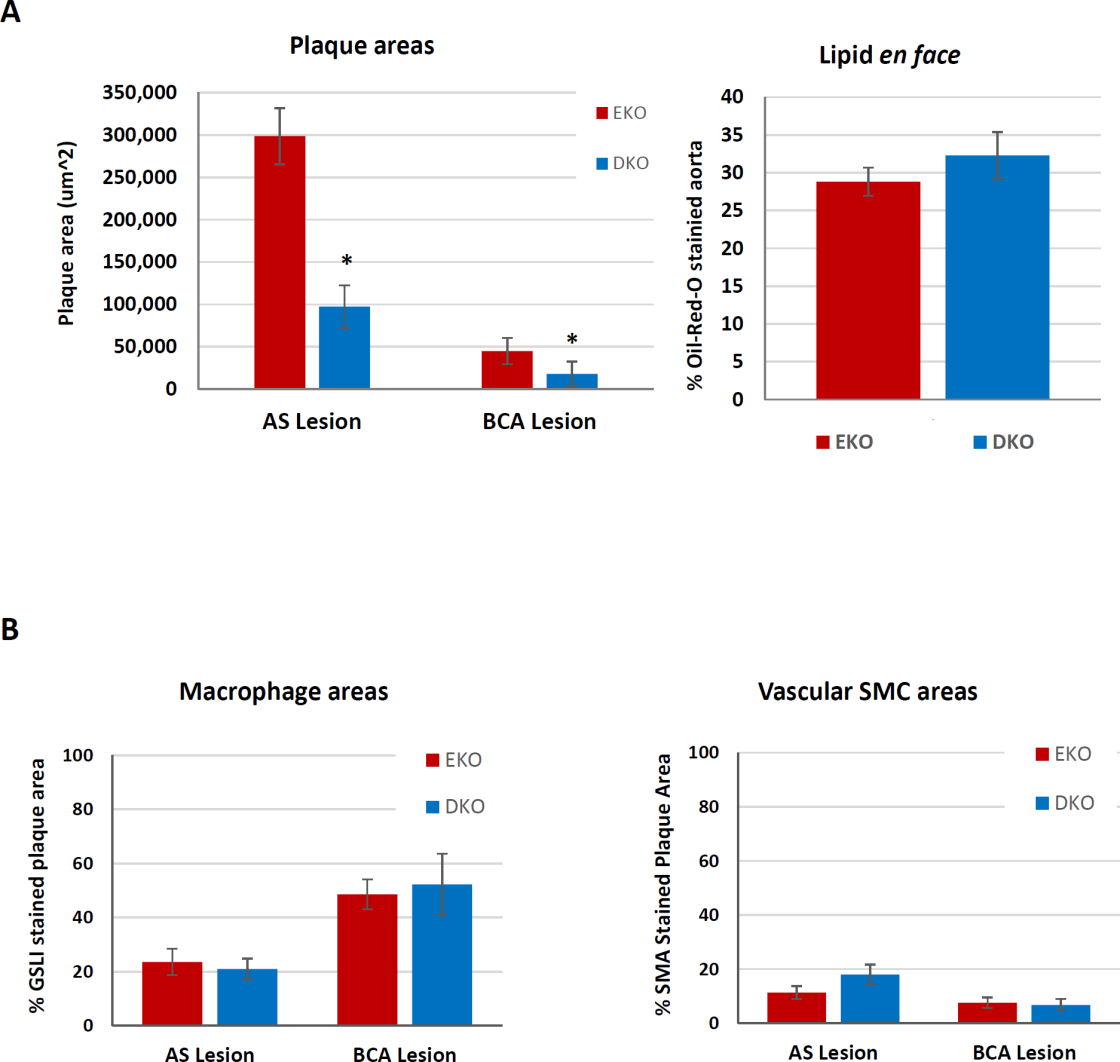
Atherosclerotic Plaque Size and composition after 35 weeks of ND in EKO vs DKO mice. Quantification of M1 and M2 markers, and MMP-s and TIMP-3 in atherosclerotic plaques of EKO and DKO mice after 35 weeks of ND. AS and BCA plaques were taken from EKO (N = 6–10) and DKO (N = 3–11) mice. Near consecutive sections were subjected to IHC for (A) NOS-2, COX-2 (as M1 markers) or Arg-1 and Ym-1 (as M2 markers) or (B) MMPs-12, -13, -14 or TIMP-3. Note that 5 of the 10 DKO mice had no plaques in the BCA. Values are expressed as mean ± SEM. * P<0.05.

When EKO mice were fed a ND for 35 weeks, lesions in the AS, BCA and aorta were approximately the same size as after 12 weeks of HFD (Fig 6A compared with Fig 3A). Both AS and BCA lesions were significantly smaller in DKO compared to EKO mice (Fig 6A), consistent with previous findings in the AS of LDLR null mice [29]. There were significantly fewer plaques in the BCA of DKO mice compared with EKO mice after 35 weeks of ND (DKO: 5/10, EKO: 9/10; Fishers Exact test, P = 0.0426). However, the areas of *en face* staining in the aorta were similar in EKO and DKO mice (Fig 6B). There was no significant difference in the percentages of AS or BCA plaque areas stained for macrophages or SMCs (Fig 6C and 6D). In addition there was no difference in the number of buried layers per BCA plaque, with EKO having 1.4 ± 0.3 and DKO having 1.5 ± 0.9 (P = 0.8769). Hence, T-bet KO reduced the size but did not affect the composition of AS or BCA plaques. AS plaques had fewer macrophages than BCA plaques irrespective of genotype (approximately 20% vs 45% of the plaque area, respectively) (Fig 6C).

### Staining for M1 and M2 markers, MMPs-12, -13 and -14 and TIMP-3

**Fig 7:**
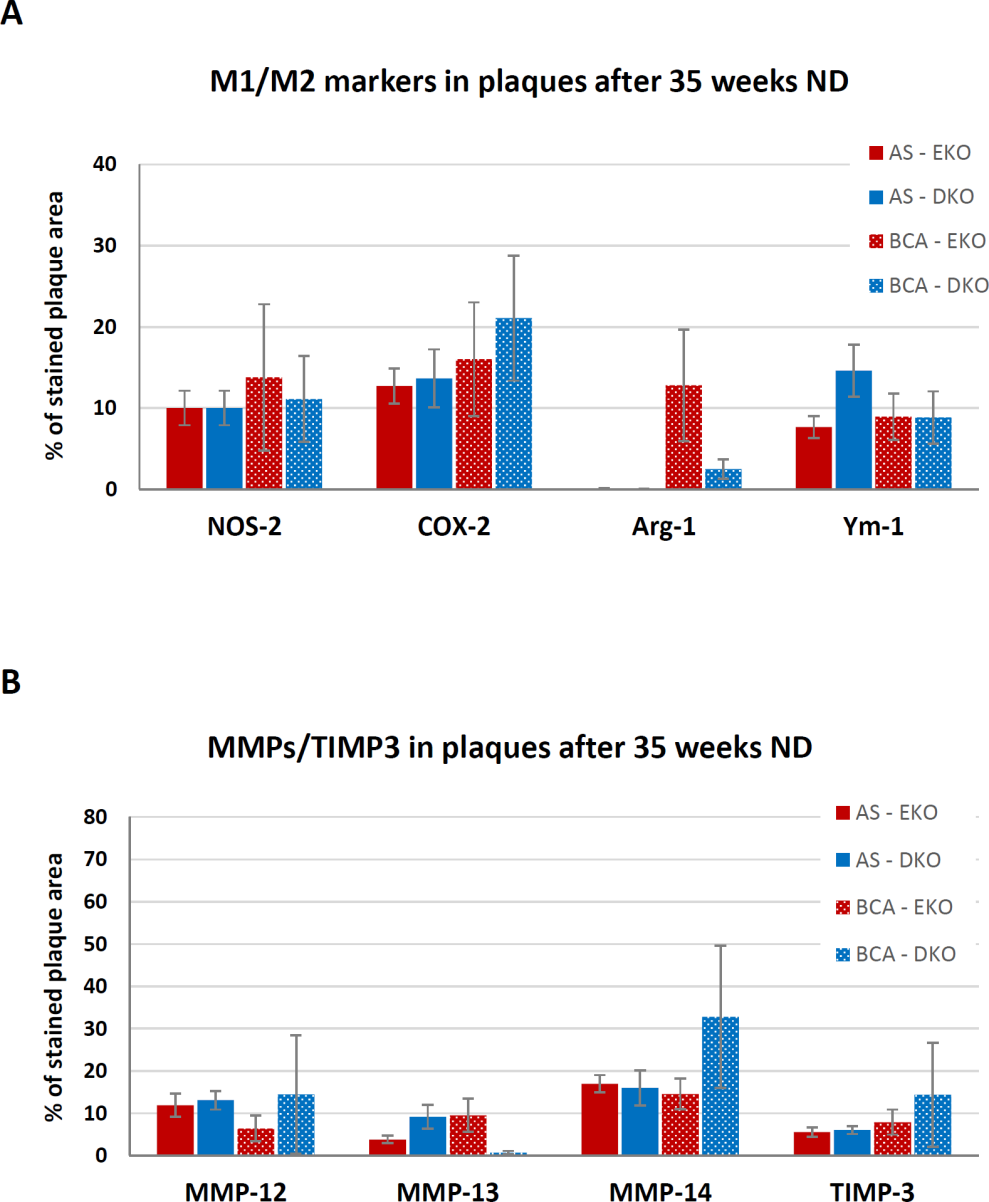
Expression of M1/M2 markers and selected MMPs/TIMP-3 in plaques of ND EKO and DKO mice. Quantification of M1 and M2 markers, and MMP-s and TIMP-3 in atherosclerotic plaques of EKO and DKO mice after 35 weeks of ND. AS and BCA plaques were taken from EKO (N = 6–10) and DKO (N = 3–11) mice. Near consecutive sections were subjected to IHC for (A) NOS-2, COX-2 (as M1 markers) or Arg-1 and Ym-1 (as M2 markers) or (B) MMPs-12, -13, -14 or TIMP-3. Note that 5 of the 10 DKO mice had no plaques in the BCA. Values are expressed as mean ± SEM. * P<0.05.

There were no significant differences in the areas of AS or BCA plaques stained by M1 or M2 markers between genotypes after 35 weeks of ND (Fig 7A). The comparison for the BCA needs caution because only five of the DKO mice had any plaque at this site. Arg-1 staining was very low in the AS of EKO or DKO mice after the 35 weeks of ND (Fig 7A). However, Ym-1 was measureable and tended to be increased in DKO mice, again providing weak evidence for increased M2 polarization (Fig 7A). Finally, there were no significant differences in staining area for MMPs-12, -13, -14 or TIMP-3 between EKO and DKO mice at either site (Fig 7B).

## Discussion

### Main findings

We show here that T-bet knockout decreases plaque size but not composition in ApoE null Bl6 mice fed a ND, but not those fed a HFD. The clear, new message from our study is that T-bet knockout has no effect on M1 macrophage polarization and only marginally increases M2 polarization on either diet. Moreover, T-bet knockout does not reduce and may even increase the mRNA expression of several MMPs that have been shown to promote plaque development and act as histological surrogates of instability. Furthermore, T-bet knockout does not increase the mRNA expression any TIMPs that have been ascribed a protective role in atherosclerosis. These results were confirmed at protein level for MMPs-12, -13 and -14, which are some of the MMPs most clearly implicated in plaque progression and instability [50] and also for TIMP-3, which has a prominent protective role [51, 52]. Given that a major consequence of T-bet deletion is preventing Th1-lymphocyte development, the implication is that reducing Th1 activity will have little effect on local macrophage activation in plaques and hence on their vulnerability to rupture.

Myocardial infarction is a prevalent cause of death worldwide, and plaque rupture is the most common mechanism [53]. Vulnerable plaques have fewer SMCs but more macrophages and T-lymphocytes than stable plaques. Furthermore, auto-reactive T-cell clones have been isolated from human plaques and this has led to the hypothesis that Th1-cell driven inflammation may exacerbate atherosclerosis and promote plaque instability [54]. More specifically, Th1 cells could stimulate macrophages towards an M1 phenotype that not only perpetuates inflammation but also directly reduces plaque stability by expressing increased amounts of extracellular proteases, including MMPs. However, this hypothesis has not until now been tested directly. Indeed, the influence of deleting Th1 cells on M1 macrophage polarization and MMP/TIMP expression has not to our knowledge been previously investigated in any model of inflammation.

### In vitro studies

As a preliminary, we investigated the regulation of a broad range of MMPs and TIMPs in mouse blood monocyte derived macrophages polarized to M1 with TNFα either in the presence or absence of IFNγ. We used high concentrations of cytokines and allowed sufficient time (18 hours) for priming effects to occur. MMPs-2, -9, and -14 were upregulated and TIMP-2 downregulated by M1 polarization with TNFα, consistent with *in vitro* observations on other preparations of mouse and human macrophages [16, 55]. MMP-13 was previously shown not to be upregulated by TNFα [56] in mouse macrophages, consistent with our present data, although M1 polarization with bacterial lipopolysaccharide was effective [16, 56]. No stimulatory effects of IFNγ were observed in our *in vitro* studies or in previous studies of mouse bone marrow derived macrophages [16]. In contrast, MMPs-12, -14 and -25 are up-regulated and TIMP-3 is greatly down-regulated by IFNγ in human monocyte derived macrophages [4]. Previous studies also indicated that, amongst the mouse genes, only MMP-19 showed significant up-regulation and none down-regulation under M2 conditions [16]. By comparison, human MMP-12, MMP-25 and TIMP-3 genes were up-regulated under M2 conditions [4]. Hence there are clear differences between mouse and human macrophages, although clear limitation of such *in vitro* studies is their inability to reflect all possible *in vivo* conditions.

### Plaque size and composition

T-bet deletion has been studied previously in atherosclerosis-prone LDLR KO mice fed a HFD [29] where a clear reduction in AS plaque size was observed in male but not female mice. For this reason we focused our own study on male mice. However, we did not observe a reduction in plaque size in DKO compared to EKO mice fed a HFD (Fig 3), and considered this might be owing to the overwhelming effect of the HFD. It was previously shown that deleting all T and B cells only reduces plaque size in EKO mice under ND and not HFD [44–46]. Consistent with this, we also observed a reduction in atherosclerosis resulting from T-bet deletion in EKO mice fed a ND (Fig 6) and this was similar in magnitude to that previously observed in LDLR KO mice [29]. Indeed the effect was quite dramatic, with half of the mice on ND having no plaque at all in the BCA. The previous studies in LDLR KO mice [29] reported no effect of T-bet deletion on the macrophage content of plaques but a decrease in SMC content, which was ascribed to the less advanced stage of these plaques. Our results also showed no difference in macrophage content of lesions of EKO and DKO mice either fed a HFD or ND. SMCs appeared much more abundant overall in EKO plaques after 12 weeks HFD compared to the reported values from LDLR KO mice [29]. We found a reduction in SMC content in the BCA but there was an increase in the AS; and no differences were found at either site after 35 weeks of ND. Hence the effects of T-bet deletion were different depending on the site and dietary conditions. Interestingly, IFNγ has been shown to stimulate [57] or inhibit [58] proliferation of SMCs in culture, depending on the conditions. By contrast, T-bet deletion significantly increased proliferation of FCMs isolated from subcutaneous granulomas, consistent with previous results showing that the Th1-cell derived cytokine, IFNγ, can inhibit macrophage proliferation [59].

### Lipid metabolism

Lower blood cholesterol levels have been observed in EKO mice fed a ND compared to a HFD continuously over 40 weeks of life [40, 48] and our observations at the time of sacrifice were consistent with these comprehensive time course studies. In their original description of the effect of T-bet deletion on LDL receptor null mice, Buono and colleagues [29] observed no difference in total circulating cholesterol levels, similar to our results. However, we also measured the HDL fraction, which was increased in DKO compared to EKO mice. In addition, Buono and colleagues [29] noted a significant reduction in total circulating IgG2a levels, and both IgG1 and IgG2a levels of antibodies to modified LDLs. Furthermore, there was an increase in serum levels of the EO6 natural antibody against phosphorylcholine. All of these factors may contribute to the decreased plaque size observed in T-bet DKO mice. Interestingly, we observed a similar increase in HDL levels in Rag-1 deleted EKO mice that lack both T- and B-lymphocytes [16]. Related effects on lipid metabolism of manipulating adaptive immunity have been recently described. For example, T-bet deletion was shown to increase visceral adiposity but paradoxically improve insulin sensitivity [60]. Furthermore, depletion of regulatory T cells in LDLR KO mice caused a variety of gene changes in the liver that led to hypercholesterolaemia [61]. One of the most prominent of these was an increase in liver sortilin expression [61] and we found a converse decrease in liver sortilin mRNA expression in DKO compared to EKO mice (results not shown). Fully defining the mechanisms underlying the complex effects of T-bet deletion on lipid metabolism would require a separate study beyond the present scope.

### Inflammation

There is a now overwhelming evidence from mouse models and humans that high fat feeding causes systemic inflammation [62]. For example, increases in several pro-inflammatory cytokines and IL-10 were observed in EKO mice fed a HFD compared to a ND for 12 weeks in a previous study [49]. The greater concentrations of the same cytokines, including IFNγ, TNFα, GM-CSF, IL-12 and IL-10, that we observed after HFD compared to ND at the point of sacrifice (Table 7 compared to Table 11) are consistent with these and other similar findings [48]. Hence, although our terminal samples were taken at different time points, the differences are likely to be due to the diets. In our experiments, HFD abolished any differences in blood cytokine levels between EKO and DKO mice, as well as any differences in mRNA expression of IFNγ and other cytokines in the liver or spleen. T-bet knockout greatly reduces release of IFNγ from CD4^+^ T-lymphocytes [29], as well as dendritic cells [28]. However, eomesodermin can still drive IFNγ production in CD8^+^ T-cells on T-bet KO mice [63] and other sources of IFNγ, including monocyte/macrophages themselves might be able to compensate for loss of Th1 cells, as has been shown directly in Rag-1 depleted mice [64], thereby replenishing plasma and tissue levels. Another inflammatory mechanism by which HFD and immunomodulation can influence atherosclerosis is through changing the amplitude of circulating monocyte populations [65], in particular increasing the mobilization of Ly6C positive monocytes from the spleen [36]. However, we did observe any differences in Ly6C levels in the spleens of our DKO compared to EKO mice.

### Macrophage polarization and MMP/TIMP expression

In T-bet null mice, it was previously shown that splenic and lymph node T-cells produce much less IFNγ and switch towards production of Th2 cytokines, including IL-4, -5 and -10 [29]. This would be expected to favour M2 polarization of macrophages in response to IL-4 and IL-10 rather than M1 polarization primed by IFNγ [66]. Our studies of peritoneal macrophages provided evidence for increased M2 polarization. However, there were no M1 polarized FCMs in the peritoneum of EKO or DKO mice, which is similar to what was observed in LDLR KO mice [38]. Our previous studies showed that FCMs obtained from subcutaneous granulomas, which are subjected to an additional stimulus from the foreign body reaction, express high levels of both M1 and M2 markers genes [16]. The present results confirmed this observation and extended it to show that, based on levels of NOS-2, T-bet deletion reduced M1 polarization. We then directly compared the levels of a large range of MMPs and TIMPs in FCMs from subcutaneous granulomas. There was not, however, any measurable reduction in the mRNA levels of MMP-2, -9 or -14 that could be increased by M1 polarization.

We extended our studies of mRNA levels to whole aortae. After 12 weeks on HFD, RT-qPCR on mRNA extracted from whole aortae showed measureable levels of all the genes we studied, but no difference between EKO and DKO mice. One could criticise these studies for analysing RNA extracted from the whole tissue, which is composed of smooth muscle and other cells as well as macrophages. We therefore investigated protein levels in the plaques directly using quantitative immunocytochemistry. We used the same markers for M1 FCMs (NOS-2 and COX-2) and M2 FCMs (Arg-1 and Ym-1) that have been validated in previous studies [16, 30, 67]. Buono and colleagues [29] found no difference in the level of expression of genes believed to be IFNγ dependent, which is consistent with our findings. Moreover, a number MMPs and TIMPs were also analysed by immunohistochemistry of plaques. MMP-12 and MMP-13 were chosen because of the strong genetic and pharmacological evidence for their involvement in mouse plaque development [34, 68–70]. MMP-14 and TIMP-3 were also studied because they appear to have opposing roles in pericellular proteolysis in macrophages, and this results in major effects on macrophage invasion, proliferation and apoptosis [71–73] as well as collagen degradation [51, 74]. It was also particularly important to measure the protein levels of MMP-14 and TIMP-3 because post-translational mechanisms control their expression [71, 72]. All of the antibodies we used gave highly specific staining. It was clear, however, that T-bet and hence Th1-lymphocytes are dispensable for M1 and M2 polarization of FCMs and for expression of MMP-12, -13, -14 and TIMP-3 in EKO mice fed a HFD. We also found no effect of T-bet deletion on M1/M2 polarization, MMP or TIMP-3 expression in the less inflammatory model of mice fed a ND for 35 weeks, despite the reduction in plaque size. Our findings and those of Buono and colleagues [29] are indicative of a systemic rather than local effect of T-bet deletion on the extent of atherosclerosis in this model.

### Summary

In summary, our data clearly show that T-bet knockout can reduce plaque size under ND but not HFD feeding. However, our main new finding is that M1 macrophage polarization and related MMP production is preserved or even enhanced in the absence of Th1 lymphocytes on either diet. The results do not encourage the deployment of strategies designed to reduce Th1 cell activation as treatments against plaque vulnerability but instead favour anti-inflammatory approaches that directly reverse M1 macrophage polarization.

## Abbreviations

ApoE: apolipoprotein E
Arg-1: arginase 1
AS: aortic sinus
BCA: brachiocephalic artery
CSF: colony stimulating factor
COX: cyclooxygenase
DAB: 3,3′- diaminobenzidine
DKO: ApoE/T-bet double knockout
EKO: ApoE knockout
FCM: foam cell macrophage
FCS: foetal calf serum
FIZZ: found in inflammatory zone
GM-CSF: granulocyte-colony stimulating factor
GSL: *Griffonia simplicifolia* lectin
Gt: goat
HBSS: Hanks’ Balanced Salt Solution
HDL: high density lipoprotein
HFD: high fat diet
IFNγ: interferon-γ
IHC: immunohistochemistry
IL: interleukin
NOS-2: inducible nitric oxide synthase
LDL: low density lipoprotein
MAb: monoclonal antibody
M-CSF: macrophage colony stimulating factor
Mm: mouse
MMP: matrix metalloproteinase
ND: normal diet
PAb: polyclonal antibody
PBS: phosphate buffered saline
PBMC: peripheral blood mononuclear cell
Rb: rabbit
RT-qPCR: real-time quantitative polymerase chain reaction
SMA: α-smooth muscle cell actin
SMC: smooth muscle cell
T-bet: T-box 21
TNFα: tumour necrosis factor-α
Th1: Thelper1
TIMP: tissue inhibitor of matrix metalloproteinase
VLDL: very low density lipoprotein
Ym1: Chil3; chitinase-like protein 3
